# Alboserpin, the Main Salivary Anticoagulant from the Disease Vector *Aedes albopictus*, Displays Anti–FXa-PAR Signaling In Vitro and In Vivo

**DOI:** 10.4049/immunohorizons.2200045

**Published:** 2022-06-23

**Authors:** Gaurav Shrivastava, Paola Carolina Valenzuela-Leon, Andrezza Campos Chagas, Olivia Kern, Karina Botello, Yixiang Zhang, Ines Martin-Martin, Markus Berger Oliveira, Lucas Tirloni, Eric Calvo

**Affiliations:** * Laboratory of Malaria and Vector Research, National Institute of Allergy and Infectious Diseases, National Institutes of Health, Rockville, MD;; † Protein Chemistry Section, Rocky Mountain Laboratories, National Institute of Allergy and Infectious Diseases, National Institutes of Health, Hamilton, MT;; ‡ Tick-Pathogen Transmission Unit, Laboratory of Bacteriology, National Institute of Allergy and Infectious Diseases, Hamilton, MT

## Abstract

Blood-feeding arthropods secrete potent salivary molecules, which include platelet aggregation inhibitors, vasodilators, and anticoagulants. Among these molecules, Alboserpin, the major salivary anticoagulant from the mosquito vector Aedes albopictus, is a specific inhibitor of the human coagulation factor Xa (FXa). In this study, we investigated the anti-inflammatory properties of Alboserpin, in vitro and in vivo. In vitro, Alboserpin inhibited FXa-induced protease-activated receptor (PAR)-1, PAR-2, PAR-3, VCAM, ICAM, and NF-**κ**B gene expression in primary dermal microvascular endothelial cells. Alboserpin also prevented FXa-stimulated ERK1/2 gene expression and subsequent inflammatory cytokine release (MCP-1, TNF-**α**, IL-6, IL-8, IL-1b, IL-18). In vivo, Alboserpin reduced paw edema induced by FXa and subsequent release of inflammatory cytokines (CCL2, MCP-1, IL-1**α**, IL-6, IL-1**β**). Alboserpin also reduced FXa-induced endothelial permeability in vitro and in vivo. These findings show that Alboserpin is a potent anti-inflammatory molecule, in vivo and in vitro, and may play a significant role in blood feeding.

## INTRODUCTION

Culicine mosquitoes are the primary vectors of many arthropod-borne (arbo) viruses around the world, including those of the Flaviviridae, Togaviridae, and Bunyaviridae families, such as Zika, dengue, chikungunya, and Rift Valley fever viruses, respectively. These viruses are the etiological agents of severe human illnesses, including hemorrhagic and biphasic fevers, encephalitis, and meningitis. They infect large populations worldwide, causing severe morbidity and mortality and a high burden of disease that could threaten half of the world’s population by the year 2050 (1–7). The global distribution of the principal vectors of these arboviruses, *Aedes* and *Culex* mosquitoes, throughout the tropics and subtropics dictates the prevalence of disease in these areas. However, globalization, climate change, and urbanization also continue to expand the range of these vectors (8, 9).

When taking a blood meal, a mosquito penetrates its proboscis into the epidermis and dermis of the skin to locate a hemorrhagic blood pool or suitable vessel. During this activity, the mosquito may release several pathogens along with its saliva at the bite site (10). Mosquito saliva contains a complex mixture of pharmacologically active compounds that facilitate blood intake by preventing host hemostasis. Hemostasis is a tightly regulated process that involves complex interactions between endothelial cells, platelets, and coagulation proteins to prevent blood loss after an injury (11). There are two main components of hemostasis: primary hemostasis, which results in local vascular contraction, thereby reducing blood flow to the injury site and formation of a weak platelet plug; and secondary hemostasis, which relies on the activation of coagulation factors that generate an insoluble fibrin clot that stabilizes the platelet plug formed during primary hemostasis. Secondary hemostasis is a complex process regulated by several proteases and coagulation factors. Factors I, II, IX, X, XI, and XII participate in the intrinsic pathway, and factors I, II, VII, and X participate in the extrinsic pathway (12). Both coagulation pathways originate separately, but they converge on a single pathway, leading to activation of factor Xa (FXa). The clotting FXa belongs to a family of serine proteases situated at the juncture of intrinsic and extrinsic pathways in the coagulation cascade and catalyzes the conversion of prothrombin to thrombin, which in turn converts fibrinogen into fibrin and plays a prominent role in various thromboembolic complications (13).

In this study, we show that Alboserpin, the main salivary anticoagulant of Aedes albopictus, displays strong anti-inflammatory activity in vivo and in vitro. Alboserpin reduced proinflammatory cytokine and chemokine release; decreased ERK1/2 expression and protease-activated receptor (PAR) signaling; and suppressed VCAM, ICAM, and NF-κb gene expression. Furthermore, Alboserpin significantly reduced paw edema in vivo and attenuated inflammatory cytokine and chemokine release. Alboserpin also inhibited the ability of FXa to disrupt endothelial permeability in vitro and in vivo.

## MATERIALS AND METHODS

### Ethics statement

Public Health Service Animal Welfare Assurance #A4149–01 guidelines were followed according to the National Institute of Allergy and Infectious Diseases (NIAID), National Institutes of Health (NIH) Office of Animal Care and Use. These studies were carried out according to the NIAID-NIH animal study protocol approved by the NIH Office of Animal Care and Use Committee, with approval ID ASP-LMVR3.

### Reagents

FXa was obtained from Hematologic Technologies. Anti-phospho-p44/42 MAPK (Erk1/2) and anti-ERK1/2 Abs were obtained from Cell Signaling Technology. GAPDH Ab was obtained from Santa Cruz Biotechnology. Secondary Abs, HRP Goat anti-Rabbit IgG and HRP Goat anti-Mouse IgG, were obtained from Invitrogen, USA and Seracare, USA, respectively.

### Cloning, expression, and purification of Alboserpin

The Alboserpin gene was codon optimized for mammalian expression and synthesized by BioBasic Inc., Markham, ON, Canada, in the VR2001-TOPO vector described in previous reports (14). Plasmid DNA was purified using NucleoBond PC 500 plasmid megaprep kits (Takara, Kusatsu, Shiga, Japan). The recombinant protein was produced by transfecting Free-Style 293F cells (Thermo Fisher) at the Protein Expression Laboratory of Leidos Biomedical Research, Inc. After 72 h posttransfection, the supernatant was collected for further protein purification experiments, as previously described in detail (15). In brief, cell supernatant containing the recombinant protein was supplemented with 500mM NaCl and 5mM imidazole and loaded onto a HiTrap Chelating column (5 ml bed volume; GE Healthcare). Fractions of the protein were obtained by passing a gradient of 25mM phosphate buffer, 500mM NaCl, 1M imidazole (pH 7.4) using an ÄKTA start purification system (GE Healthcare). The protein fractions were visualized by NuPAGE SDS gel (Thermo Fisher Scientific). The fractions of interest were pooled and concentrated using Amicon Ultra-15 (10-kDa cutoff) centrifugal filter units (Millipore Sigma) before the last step of purification using size exclusion chromatography (Superdex 200 10/300 GL column; Cytiva). Purified protein (Supplemental Fig. 1A, 1B) was further processed to remove any LPS contaminant and verified using Endosafe nexgen-PTS (Charles River Laboratories) described in a previous report (16). The identity of the purified protein was confirmed by N-terminal sequencing.

### Production of anti-Alboserpin polyclonal Abs and quantification of Alboserpin in salivary gland extracts

Polyclonal Abs against Alboserpin were raised in rabbits by Noble Life Sciences (Woodbine) using a standard protocol. Rabbits were immunized three times with 125 μg of Alboserpin every 21 d, and the serum was collected at day 72. A 10-ml aliquot of rabbit serum was diluted to 50 ml in phosphate buffer (pH 6.5) and loaded onto a 5-ml HiTrap Protein A HP column (GE Healthcare Life Science) and the IgG eluted with a linear gradient of citric acid (0–1 M, pH 3.4) using an Akta purifier system (GE Healthcare Life Science). Fractions containing purified IgG were pooled and dialyzed against 1× PBS for 16 h at 4°C.

Salivary gland homogenates from 3- to 5-d-old adult female *A*. *albopictus* (pools of five pairs) were diluted in 100 μl of PBS and immobilized overnight at 4°C on a 96-well, flat-bottom plate (Costar, Corning). Wells were washed with PBS and blocked for 2 h with BSA (2% v/v, in PBS). Wells were washed three times in PBST (0.05% v/v Tween 20) and incubated with rabbit anti-Alboserpin (10 μg/ml) in PBST supplemented with 0.05% (v/v) BSA for 2 h. Wells were washed three times with PBST and incubated with alkaline phosphatas–coupled anti-rabbit IgG (1:10,000 in PBST; Sigma). After 1-h incubation, the wells were washed four times with PBST, and the stabilized p-nitrophenyl phosphate liquid substrate (Sigma) was added. Colorimetric analysis was performed by measuring absorbance values at 405 nm in a VERSAmax microplate reader (Molecular Devices). Standards (0.01–1000 ng of recombinant Alboserpin) were fitted by using a nonlinear four-parameter regression to generate a standard curve. Alboserpin concentration in salivary gland extract was calculated using the standard curve.

### Cell culture

Primary Dermal Microvascular Endothelial Cells; Normal, Human, Neonatal (HDMVECns) were maintained in Vascular Cell Basal Medium (American Type Culture Collection [ATCC]) supplemented with Microvascular Endothelial Cell Growth Kit-VEGF (ATCC) in a 5% CO_2_–air mixture at 37°C. Subconfluent cultures were washed twice with PBS (pH 7.4; Life Technologies), and cells were detached with Trypsin-EDTA (ATCC) and neutralized with Trypsin Neutralizing solution (ATCC). Cells were seeded at 5 × 10^5^ cells per well in six-well tissue culture plates and further treated with either FXa (50 nM), Alboserpin (50 nM), or FXa + Alboserpin (50:50 nM) for 3 h.

### FITC-dextran permeability assay

HDMVECns obtained from ATCC were seeded (40,000 cells/Transwell insert) on 1% gelatin-coated Transwell inserts (24 wells, 0.4 mm, 6.5-mm insert; Corning Inc.) for a minimum of 4 d and maintained according to the manufacturer’s instructions, until a confluent cell monolayer formed. Initially, the medium was replaced with a basal medium at 3% FBS (Life Technologies) with 50 nM Alboserpin, along with FITC-dextran (70-kDa, 1 mg/ml; Sigma), and incubated for 30 min in the upper chamber. Further, the upper chamber was either treated with 50 nM FXa or left untreated along with FITC-dextran. Cells treated with FXa alone were used as a positive control. All the conditions were incubated for 30, 60, 120, and 240 min at 37°C at 5% CO_2_ and 90% humidity. Transwell inserts were removed at every time point, and 50 μl from each well was collected from duplicate wells and transferred to a black 96-well flat-bottom plate (Costar). The amount of FITC-dextran that migrated from the upper to the lower chamber was used to evaluate the endothelial permeability. FITC-dextran fluorescence was measured at an excitation wavelength of 485 nm and emission wavelength of 528 nm using the Cytation 5 reader (BioTek Instruments). Alteration of membrane permeability was detected by crystal violet stain. In brief, the cells were fixed with 4% paraformaldehyde for 15 min, stained with 1% crystal violet for 30 min, and finally washed three times with 1× PBS. Images were captured with the Echo Revolve Microscope (Echo Laboratories).

### Western blot analysis

Whole-cell extracts were isolated using Pierce RIPA Buffer (Thermo Scientific) supplemented with protease and phosphatase mixture inhibitor (Abcam). The concentration of the isolated proteins was determined using the DC Protein Assay kit II (Bio-Rad). Thirty micrograms of protein from whole-cell lysates was separated on NuPAGE 4–12% Bis-Tris gels (Invitrogen). Proteins were transferred to a nitrocellulose membrane (iBlot; Invitrogen) and then blocked for 1 h at 37°C with blocking buffer (5% [w/v] powdered nonfat milk in TBST). Polyclonal Abs against Phospho-p44/42 MAPK (Erk1/2) and ERK1/2 (Cell Signaling, USA) were diluted in blocking buffer (1:1000) and incubated overnight at 4°C. As a loading control, a mAb against GAPDH (Santa Cruz) was diluted in blocking buffer (1:1000), and the blot was incubated for 1 h at 4°C. HRP Goat anti-Rabbit IgG (Invitrogen) and HRP Goat anti-Mouse IgG (Invitrogen), diluted in blocking buffer at 1:5000 and 1:3000, respectively, were used as secondary Abs. Immunogenic bands were developed with SuperSignal West Femto Maximum Sensitivity Substrate (Thermo Scientific). Blots were imaged using an Azure 300 imaging system (Azure Biosystems).

### In-Cell Western blot

Phospho-ERK1/2 and ERK1/2 protein expression were assessed using In-Cell Western analysis. In brief, HDMVECns were seeded in a black 96-well plate (2 × 10^3^ cells/well) and cultured in endothelial media (Vascular Cell Basal Medium, complemented with Endothelial Cell Growth Kit-BBE; ATCC) for 1 d. At the end of the treatment, cells were immediately fixed in 4% cold paraformaldehyde for 20 min at room temperature (RT), then washed five times with 0.1% Triton X-100 in PBS. The plates were blocked with Azure fluorescent blocking buffer (Azure Biosystems) for 60 min at RT with moderate shaking followed by incubation with Phospho-p44/42 MAPK (Erk1/2) and ERK1/2 Abs (1:300 dilution) with Azure fluorescent blocking buffer overnight at 4°C. The plates were washed five times with 0.1% Tween 20 in PBS and gently agitated for 5 min at RT. Next, secondary Abs (Azure secondary Ab, anti-Rabbit 700) were added at a 1:200 dilution to each well and incubated in the dark for 1 h at RT with gentle shaking. The plates were washed five times with 0.1% Tween 20 in PBS and gently agitated for 5 min at RT. Finally, the plates were scanned at 700 and 800 nm, and the intensity of the labeled proteins was measured using the Azure sapphire biomolecular imager (Azure Biosystems). Each experiment was performed in duplicate. Negative controls were obtained by omitting the primary Ab during the incubation steps, and background values were obtained by omitting primary and secondary Abs.

### FXa cleavage of PAR-2 peptide

The cleavage activity of FXa (Enzyme Research) on PAR-2 peptide was investigated based on fluorescence resonance energy transfer technology. Peptide sequence that spans the cleavage sites for mouse PAR-2 (NSKGRSLIGR) was synthesized with the fluorescent group 7-methoxycoumarin-4-acetic acid and the quenching group Dnp (2,4-DNP) at the N- and C-terminal ends, respectively (Anaspec). FXa (10 nM) was incubated with different concentrations of purified Alboserpin (0–200 nM) at 37°C in 20 mM Tris–HCl, 150 mM NaCl, Tween 20 0.01% (pH 7.4). After 15-min incubation, peptide was added in a 100 μl final reaction volume. The peptide hydrolysis rate was followed at 320 nm excitation and 420 nm emission in kinetic mode at 30°C in a Synergy H1 max microplate reader (Biotek Instruments).

### Electrospray ionization-mass spectrometry analysis of FXa-digested PAR-2 peptide

A Q Exactive plus (Thermo Fisher Scientific) was used to carry out electrospray ionization-mass spectrometry experiments. The mass spectrometer was operated at resolution 280,000, spray voltage 3.5 kV, and capillary temperature 320°C. Samples were desalted by C18 Zip-tip (Millipore) and dissolved in 100 μl of reconstitution buffer (50% ACN, 49% H_2_O, 1% FA). Samples were introduced using a syringe pump (Thermo Fisher Scientific) with a flow rate of 5 μl/min. Xtract algorithm of the Freestyle (Thermo Fischer Scientific, version 1.5) was used to deconvolute the raw data. Deconvolution parameters were set as: output mass, MH+; charge range, 1–5; minimum number detected charge, 2.

### Gene expression analysis

HDMVECns treated with experimental conditions were collected in TRIzol (Life Technology) and stored at −80°C. Total RNA was extracted with TRIzol, according to the manufacturer’s instructions (Life Technologies). cDNA was prepared using the LunaScript RT SuperMix Kit (New England Biolabs), from 1 μg of extracted RNA from three technical replicates. All nucleic acid concentrations and OD 260/280 ratios were measured by a DS-11 spectrophotometer (DeNovix). For quantitative PCR (qPCR), the specific primers were designed and used (Supplemental Table I). In brief, in a final volume of 20 μl, the reaction mixture was prepared with Luna Universal qPCR Master Mix (New England Biolabs), 300 nM of each forward and reverse primer, and 10 ng of cDNA template. Reactions were run on a CFX96 thermocycler (Bio-Rad) using the following amplification protocol: 95°C, 2 min; 40 cycles of 95°C, 15 s; 60°C, 30 s. A melting curve (60°C–95°C) was initially included to evaluate primer specificity. All samples were analyzed in technical triplicate, and nontemplate controls were included as negative controls. qPCR data were manually examined and analyzed using the ΔΔCt method. ΔCt values were obtained by normalizing the data against the GAPDH housekeeping gene primer (Supplemental Table I). Untreated cell samples were used as ΔΔCt controls. The relative abundance of genes of interest, or fold change, with respect to the untreated cells was calculated as 2^ΔΔct^. Graphs were prepared using GraphPad Prism software version 9.0.

### Paw edema and cytokine measurements

Eight-week-old female C3H/HeJ mice were used (Charles River Laboratories) and maintained in the NIAID Animal Care Facility. Posterior footpads were injected with PBS, Alboserpin (1.5 μg), FXa alone (1.5 μg), or FXa incubated with Alboserpin (1.5 mg + 1.5 mg) for 15 min. Intradermal/s.c. injections were performed by injecting 50 μl of sample into the footpads using BD Microfine IV needles. The thickness of the posterior footpad was recorded using a caliper (Mitutoyo America Corp., Kawasaki, Kanagawa, Japan) before each injection and at 15, 30, 45, and 60 min. After the last measurement, animals were euthanized, and footpads were removed. Footpads were cut into three to four pieces with a blade and added to gentleMACS C Tubes (Miltenyi Biotec, Bergisch Gladbach, Germany) containing 1 ml of PBS. Tissues were homogenized using a multitissue program (multiA) from the gentleMACS Dissociator (Miltenyi Biotec). After centrifugation at 4000 rpm for 10 min at 4°C, supernatants were filtered using a Millex-GV Filter, 0.22 mm, polyvinylidene difluoride, 13 mm, ethylene oxide sterilized (Millipore). Filtered supernatants were stored at −80°C until processing for cytokine measurements using Mouse Magnetic Luminex Assay kit (R&D Systems) and Luminex 200 machine, according to the manufacturer’s instructions. Two biological replicates with a total of eight animals were used in these experiments.

### Miles vascular permeability assay

A Miles assay using Evans blue dye (National Aniline Division) is a conventional method to assess vascular permeability in vivo (17, 18). Female BALB/c mice (6–7 wk old) were shaved 1 d before the assay. Twenty-four hours later, mice were anesthetized by inhalational isoflurane (furane 4%) and retro-orbitally injected with 50 μl of 1% Evans blue dye. After allowing Evans blue to circulate in the vasculature for 20 min, intradermal injection with 20 μl of the following substances was performed: Alboserpin (1.5 μg), FXa (1.5 μg), and a mix of Alboserpin and FXa at the same concentration, in a 1:1 proportion. PBS injections (20 μl) were used as a negative control. After 2 h, the skin biopsies were dissected and dried overnight at 56°C, and the dye was then eluted from the dissected samples with 250 μl formamide at 56°C for 12 h. Skin areas were carefully cut using a punch biopsy (diameter size, 3 mm) to ensure that the same skin size was included for all samples. The amount of Evans blue extracted from the skin samples was measured by spectrophotometry at 620 nm using a VersaMax Microplate Reader (Molecular Devices).

### Statistical analysis

Results are expressed as mean ± SEM. Statistical differences among treatment groups were analyzed by *t* test or one-way ANOVA using Tukey or Dunnett as a test formultiple comparisons. Significance was set at *p* ≤ 0.05 (GraphPad Prism software).

## RESULTS

### Quantification of Alboserpin in salivary gland extracts of *A. albopictus* mosquitoes

Recombinant Alboserpin was produced for functional and biochemical characterization studies (Supplemental Fig. 1A, 1B) as described before (19). The identity of purified recombinant protein was confirmed by N-terminal sequencing. Polyclonal Abs against Alboserpin were raised in rabbits and used for Western blot and ELISA analysis. Purified Alboserpin and a single band from salivary gland extracts migrated at similar m.w. in the immunoblot (Supplemental Fig. 1C). The amount of Alboserpin present in salivary gland extract of female mosquitoes was assessed by ELISA. We found that the concentration of Alboserpin is 6.23 ± 0.11 ng/pair. It has been shown that mosquito secreted >50% of the total salivary gland content during blood feeding (20), and that the volume of blood meal at the bite site is estimated to be between 2 and 3 μl (mosquitoes can take 1 and 5 μl of blood), then Alboserpin can be at a high enough molar concentration (10–15 nM) to inhibit FXa at the bite site. Interestingly, Alboserpin has high affinity for phosphatidylcholine:phosphatidylserine vesicles and heparin. Alboserpin could conceivably use heparin and phosphatidylcholine:phosphatidylserine as anchors to increase protein localization and achieve higher concentration at sites of injury, cell activation, or inflammation (19). For all experiments in this work, the concentration of Alboserpin was chosen based on the stoichiometry of the Alboserpin-FXa reaction, calculated to be 1:1. The amount of FXa used for the experiments was based on published data (21) with a 5-fold molar excess of Alboserpin.

### Alboserpin displays anti-inflammatory activity in vitro

**FIGURE 1.**
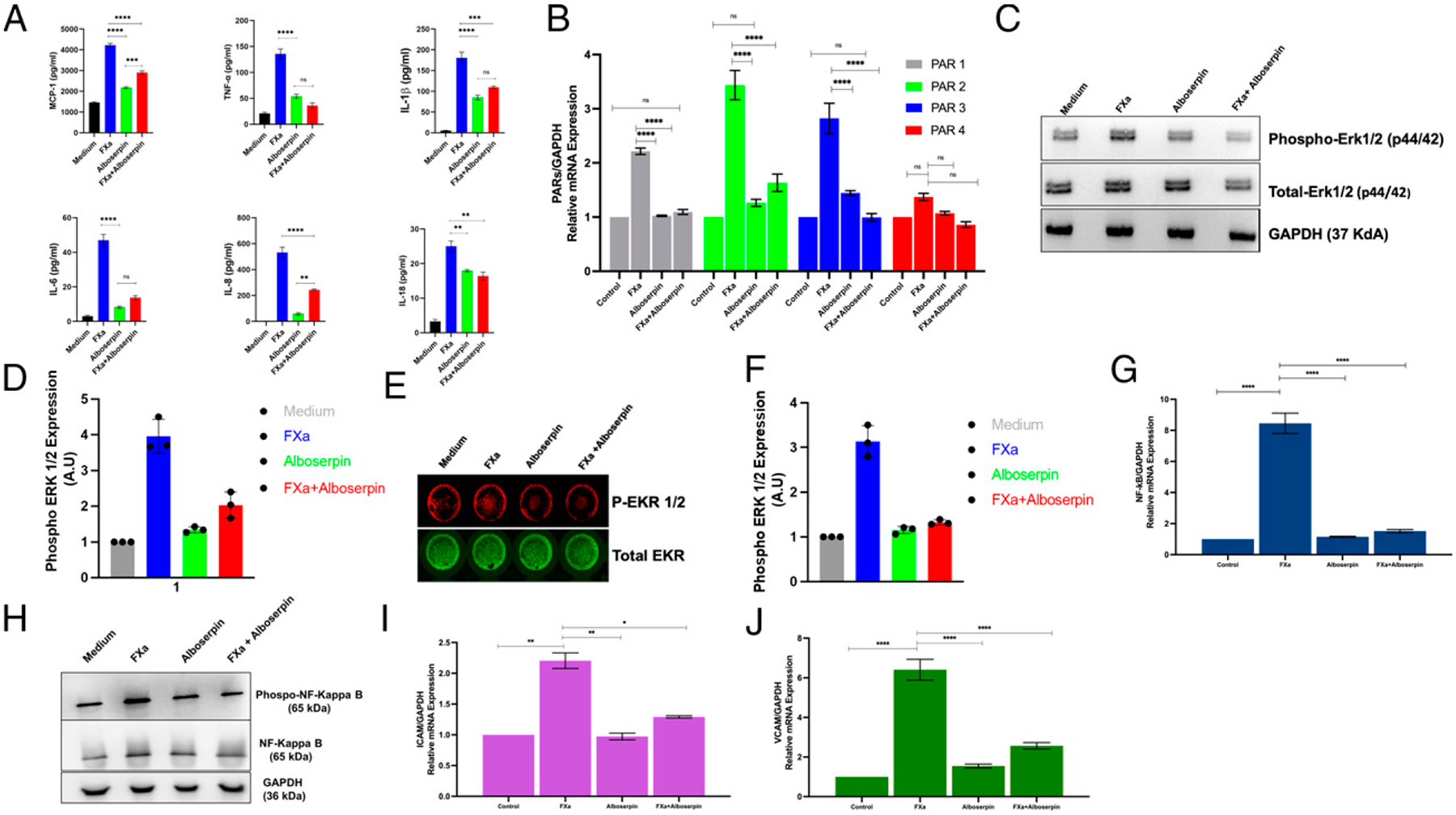
Alboserpin inhibits FXa inflammatory effect in vitro. HDMVECns were left untreated (control), treated with Alboserpin (50 nM) or FXa (50 nM), or Alboserpin:FXa for 3 h. **(A)** Cell supernatants were used for cytokine and chemokine Luminex ELISA. **(B)** Gene expression of PARs in HDMVECns. Data are shown as relative quantification versus control (resting cells). **(C)** ERK1/2 protein expression was analyzed by Western blot. Cell lysates were analyzed for levels of p-ERK, and ERK (total ERK) and GAPDH was used as a loading control. (**D**) The ratio of p-ERK/ERK is presented in arbitrary units (A.U). (**E**) ERK1/2 protein expression was analyzed using In-Cell Western blots (ICW). (**F**) Intensity ratio (p-ERK/ERK). (**G** and **H**) NF-κB gene expression and protein expression were analyzed using RT-PCR and Western blots, respectively. (I and J) ICAM and VCAM gene expression were analyzed using RT-PCR. Data are shown as relative quantification versus control (resting cells). Data from three independent experiments performed in triplicate are plotted. Error bars indicate SEM. *p < 0.05, **p < 0.01, ***p < 0.001, ****p < 0.0001.

FXa, a key component of the coagulation cascade, also triggers inflammation by interacting with PARs. PARs are integral membrane proteins that are coupled to G proteins and are activated by FXa cleavage of the amino-terminal sequence that exposes a new N-terminal sequence that functions as a tethered ligand, which then bind a conserved region on extracellular loop 2. Such binding causes the specific change in conformation of the PAR and alters the affinity for intracellular G protein, which initiates cell signaling, leading to the expression of adhesion molecules, procoagulant tissue factor, and production of proinflammatory cytokines (22–24). To evaluate the ability of Alboserpin to block FXa-induced PARs activation in vitro, we preincubated HDMVECns with Alboserpin and further stimulated them with FXa, and cytokine release was measured using a Luminex cytokine ELISA kit. Alboserpin significantly reduced FXa-induced release of proinflammatory cytokines MCP-1, TNF-α, IL-1β, IL-6, IL-8, and IL-18 (Fig. 1A). Alboserpin alone had no significant effect on cytokine release.

FXa triggers the PAR-1–, PAR-2–, and PAR-3–dependent cell signaling pathway in a cell-type– and cofactor-specific manner. Studies in lung fibroblast and endothelial cells showed that the release of FXa-induced cytokine production was due to PARs activation (25, 26). However, in RAW 264.7 macrophages and human vascular smooth muscle cells, FXa induced the expression of proinflammatory cytokines via PAR-2 activation (27, 28). In dermal endothelial cells and fibroblasts, PAR-1 and PAR-2 trigger FXa-induced secretion of cytokines. Several studies have also indicated the proinflammatory reactions because of multiple serine proteases by PAR-2 (29, 30).

To further investigate the different responses of FXa in HDMVECns, we measured PAR-1, PAR-2, PAR-3, and PAR-4 mRNA levels by qPCR. Analysis revealed a 2- to 2.5-fold increase in PAR-1 expression, a 3- to 4-fold increase in PAR-2 expression, and a 2.5- to 3.5-fold increase in PAR-3 expression in HDMVECns after FXa stimulation. However, no differences among treatment were found for PAR-4. Alboserpin alone did not change any PAR expression levels, demonstrating the role of Alboserpin as a potent inhibitor of PAR-1, PAR-2, and PAR-3, mediated by FXa stimulation (Fig. 1B). Cells treated with FXa increased p-ERK1/2 expression after 1 h. However, in the presence of Alboserpin, FXa did not produce p-ERK1/2. Alboserpin alone had no effect on p-ERK expression (Fig. 1C, 1D). To support the Western blot results, we performed In-Cell Western blot under the same conditions. In-Cell Western analysis confirmed that Alboserpin inhibited p-ERK1/2 expression in FXa-treated cells (Fig. 1E, 1F). These results demonstrate the inhibitory activity of Alboserpin by reducing FXa-induced ERK1/2 signaling.

Activation of PAR-2 by FXa also activates the transcription factor NF-κB, leading to proinflammatory upstream coagulation protease signaling responses (31–34). We measured NF-κB mRNA levels by qRT-PCR in HDMVECns treated with FXa. Adding Alboserpin significantly suppressed NF-κB gene expression triggered by FXa. Alboserpin alone did not change NF-κB expression levels in HDMVECns, demonstrating Alboserpin’s role as a potent inhibitor of NF-κB–mediated transcription stimulated by FXa cells (Fig. 1G). We also evaluated the phosphorylation of kinases in the NF-κB signaling pathway/cascade after treatment with FXa in the presence or absence of Alboserpin. FXa alone induced a significant increase in NF-κBp65 phosphorylation at Ser536, whereas FXa-Alboserpin blocked NF-κB activation (Fig. 1H).

FXa also triggers expression of the adhesion molecules ICAM and VCAM-1, as well as production of the proinflammatory cytokines IL-6, IL-8, and MCP-1 in human endothelial cells (35). To assess the effect of Alboserpin on the FXa-induced expression of the adhesion molecules ICAM and VCAM-1, we measured changes in gene expression levels after Alboserpin treatment. qRT-PCR analysis revealed a 2- to 2.5-fold increased expression of ICAM and a 6- to 7-fold increased expression of VCAM in HDMVECns after FXa stimulation. The addition of Alboserpin suppressed ICAM and VCAM gene expression triggered by FXa. Alboserpin alone did not change either ICAM or VCAM expression levels, demonstrating the role of Alboserpin as a potent inhibitor of FXa-mediated ICAM and VCAM expression (Fig. 1I, 1J). Furthermore, activation of ERK1/2 and NF-κB signaling in response to FXa resulted in an increased expression of the proinflammatory molecules ICAM and VCAM. When Alboserpin was added, expression of ERK1/2, NF-κB, ICAM, and VCAM triggered by FXa was significantly reduced (Fig. 1C–J).

### Alboserpin inhibits FXa cleavage of PAR-2 peptide

**FIGURE 2.**
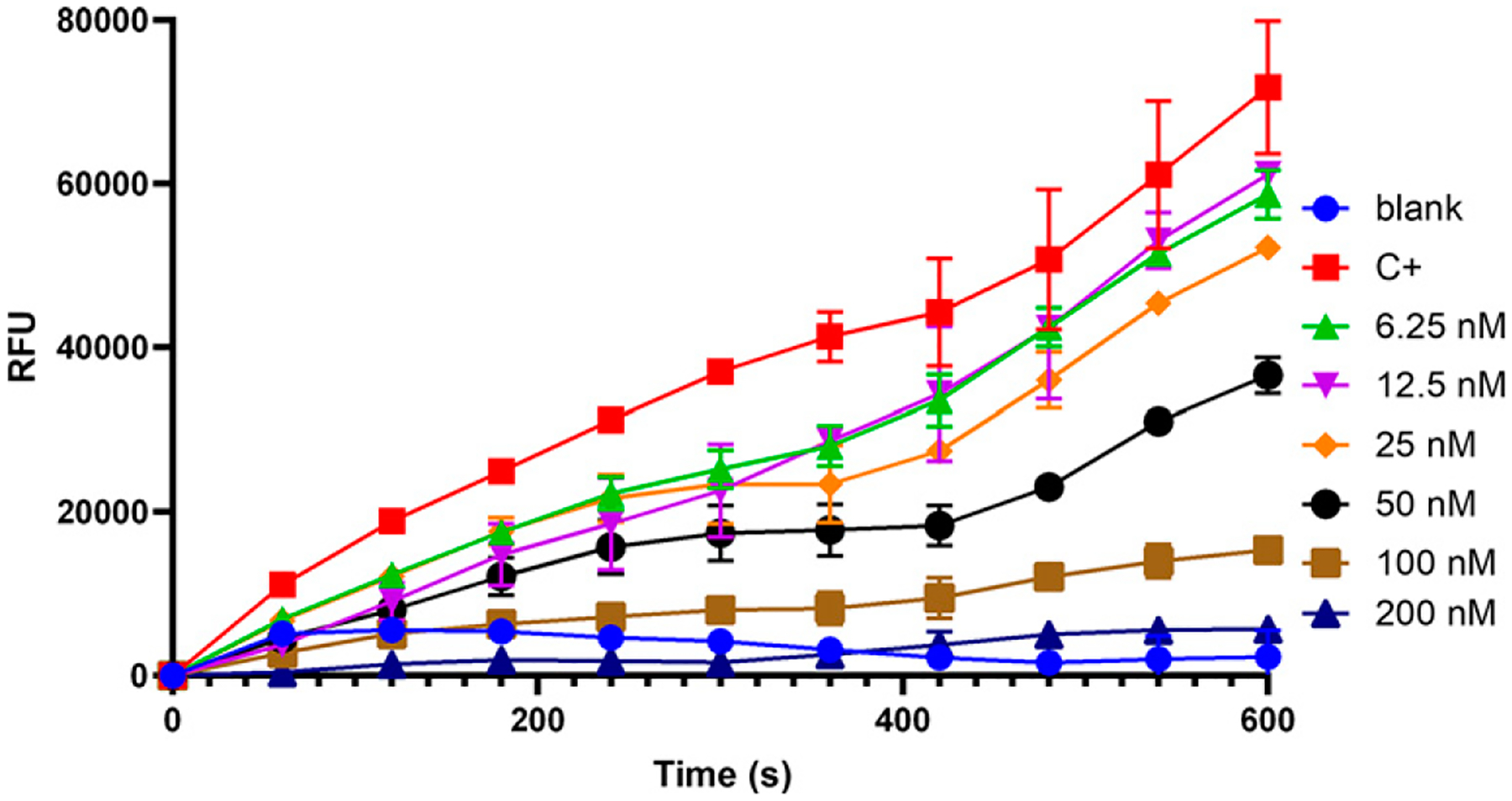
Alboserpin inhibits cleavage of PAR-2 peptide by coagulation of FXa. Representative data of FXa cleavage on PAR-2 peptide was investigated based on fluorescence resonance energy transfer (FRET) technology. Peptide sequence that spans the cleavage sites for mouse PAR-2 (NSKGRSLIGR) was synthesized with the fluorescent group Mca (7-methoxycoumarin-4-acetic acid) and the quenching group Dnp (2,4-DNP) at the N- and C-terminal ends, respectively. FXa (10 nM) was incubated with different concentrations of purified Alboserpin (0–200 nM) at 37°C in 20mMTris–HCl, 150 mM NaCl, Tween 20 0.01% (pH 7.4). After 15-min incubation, peptide was added in a 100 μl final reaction volume. The peptide hydrolysis rate was followed at 320 nm excitation and 420 nm emission in kinetic mode at 30°C in a microplate reader

PARs are integral membrane proteins coupled to G proteins and are activated by cleavage of the amino-terminal sequence that exposes a new N-terminal sequence that functions as a tethered ligand, which then bind a conserved region on extracellular loop 2 (36). PARs can be activated by the action of serine proteases, including FXa. Because Alboserpin is a specific inhibitor of FXa (19), we hypothesized that inhibition of FXa by Alboserpin leads to the inability of this protease to activate PAR. Our results (Fig. 2) show that, in the presence of Alboserpin, FXa is unable to cleave PAR-2 in vitro. We also confirmed these results by mass spectrometry analysis of PAR-2 peptide treated with FXa in the presence or absence of Alboserpin (Supplemental Fig. 2). These results demonstrate the mechanism of action of Alboserpin on FXa-induced PAR signaling.

### Alboserpin inhibits FXa-mediated acute inflammation in vivo

**FIGURE 3.**
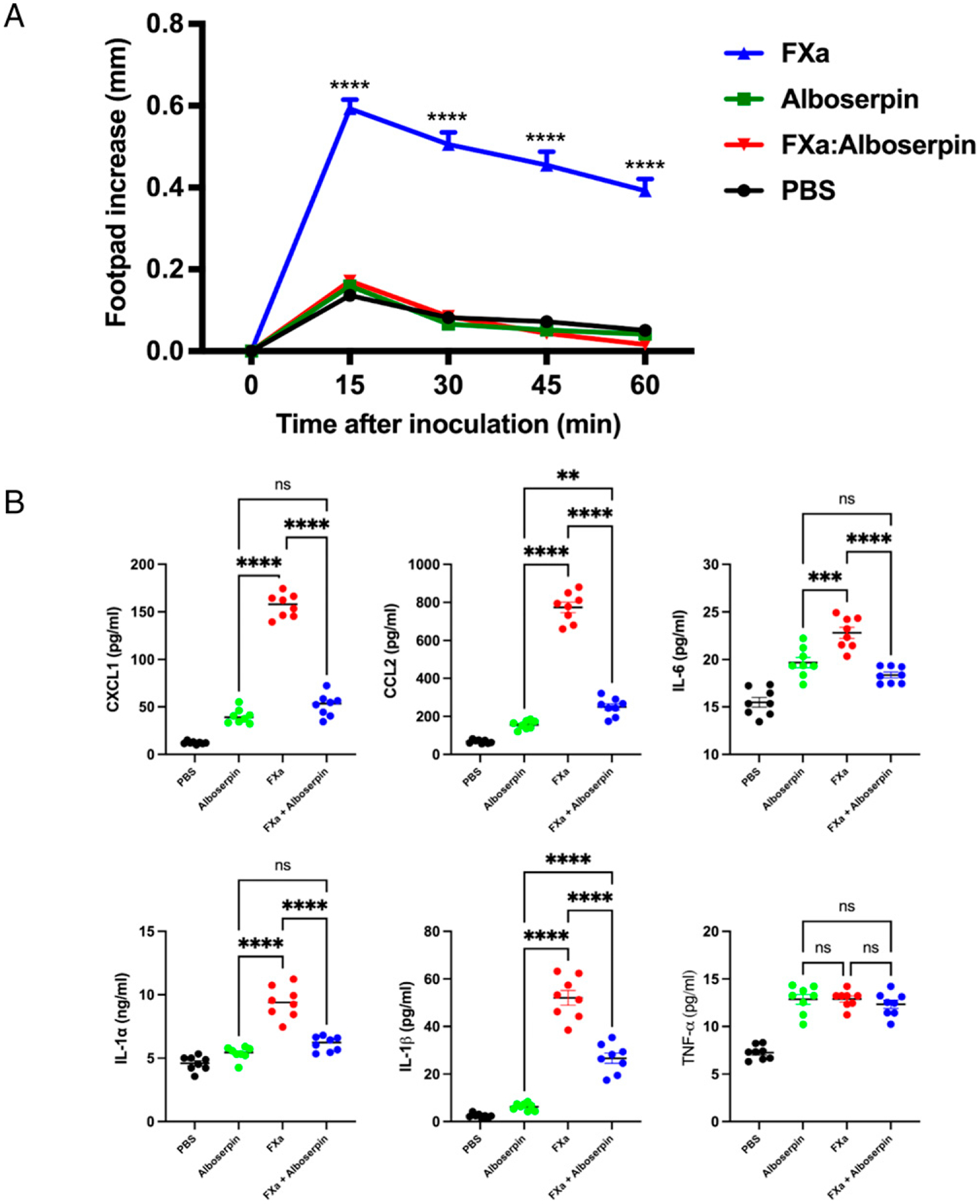
Alboserpin inhibits FXa-induced inflammation in vivo. Effect of Alboserpin in paw edema assay triggered by FXa. (**A**) Footpads of C3H/HeJ mice were intradermally injected with 30 μl of FXa (50 nM) alone or incubated along with 50 nM Alboserpin. As a control, 30 μl of PBS or 30 ml of 1 mg Alboserpin was injected. Formation of edema (increasein paw thickness in millimeters) was measured using a caliper before injection of FXa or after 15, 30, 45, and 60 min of injections. Data represent SEM of five footpads per experimental group. (**B**) Effect of Alboserpin in FXa-induced inflammatory cytokine and chemokine. Cytokine and chemokine levels in paw tissue extracts after 60 min of intradermal injection were determined by Luminex ELISA assay. Data from three independent experiments performed in triplicate are plotted. Error bars indicate SEM of eight footpads per experimental group. Error bars indicate SEM. **p < 0.01, ****p < 0.0001. ns, not significant.

Previous studies have shown that FXa subplantar injection induces time- and dose-dependent edema in the footbeds of mice and rats, and the observed effects were a result of PAR-2 activation (21, 37). To assess whether Alboserpin also inhibits local inflammatory activity of FXa in vivo, we used a mouse paw edema experiment. Inoculation of FXa triggered a time-dependent increase in paw edema that reached a maximum at 15 min and returned to the basal level after 1 h (37). Premixed FXa:Alboserpin (1:1 molar ratio) significantly reduced paw edema, demonstrating the potent anti-inflammatory activity of Alboserpin in vivo. Alboserpin alone and PBS had no effect on paw edema formation (Fig. 3A).

We also measured expression of inflammatory cytokines and chemokines from mouse paws. Fig. 3B shows that FXa enhanced the release of CXCL1, CCL2, IL-6, IL-1μ, IL-1β, and TNF-α. When Alboserpin was administered with FXa, expression of proinflammatory mediators CXCL1, CCL2, IL-1α, IL-1 b, and IL-6 was significantly reduced. No effect was observed in TNF-a secretion. Alboserpin alone had no significant effect on cytokine and chemokine secretion.

### Alboserpin reduces FXa-induced endothelial permeability in vitro and in vivo

**FIGURE 4.**
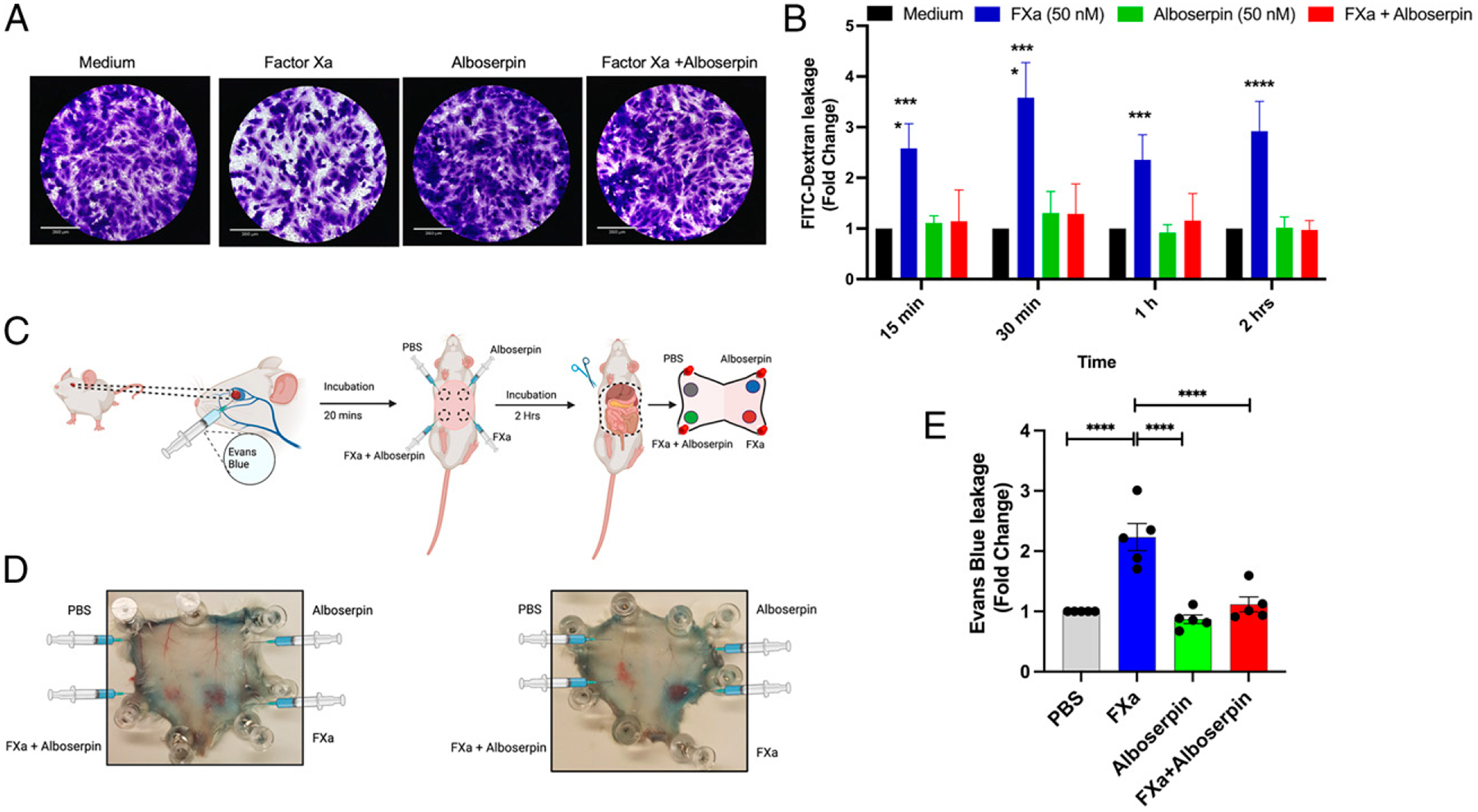
Alboserpin inhibits FXa-induced endothelial permeability disruption in vitro and in vivo. Endothelial cell monolayer permeability assay using FITC-dextran. (**A**) Crystal violet staining of the HDMVECn monolayer pretreated with Alboserpin and FXa at 50 nM for 4 h. (**B**) The fluorescence intensity of FITC-conjugated dextran leaking from the upper to the lower chambers was measured at different time points after treatment of HDMVECns with either FXa alone, Alboserpin, or Alboserpin along with FXa. Data from three independent experiments performed in duplicate are plotted. Error bars indicate SEM. (**C** and **D**) Schematic representation of the sequential steps of the Miles assay in the mouse and corresponding inoculations in different locations in skin biopsies. (**E**) Spectrophotometric analysis (610 nm) of vascular leaked formamide-extracted Evans blue dye content in the skin injected with 50 μM Alboserpin, FXa, and both FXa-Alboserpin at the same concentration. PBS was used as a negative control. *p < 0.05, ***p < 0.001, ****p < 0.0001. ns, not significant.

Endothelial cell barrier permeability at the blood–tissue interface has a significant role in inflammatory disorders such as sepsis (38). FXa disrupts the endothelial barrier in vitro by activating PAR-1 and PAR-2. Other PARs inhibitors, along with FXa inhibitor (rivaroxaban), protect the endothelial barrier in response to FXa treatment (39, 40). Because Alboserpin is a specific, tight-binding inhibitor of FXa (19), we evaluated the role of Alboserpin in endothelial permeability disruption. In vitro vascular permeability assays showed that cells pretreated with FXa had significant endothelial permeability disruption compared with the cells left untreated or treated with Alboserpin alone (Fig. 4A, 4B). When Alboserpin was premixed with FXa, cell permeability was significantly reduced (Fig. 4A, 4B). These results demonstrated prevention of endothelial cell barrier disruption in cultured cells.

We also investigated the effect of Alboserpin on endothelial permeability in vivo. BALC/c mice were retro-orbitally injected with Evans blue and then inoculated intradermally with FXa, Alboserpin, or Alboserpin-FXa (1:1 molar ratio) (Fig. 4C). Two representative skin biopsies that were taken 2 h after injection are shown in Fig. 4D. A reduction of vascular leakage was observed in the skin biopsies inoculated with FXa:Alboserpin mixture, when compared with FXa alone. Dye quantification of skin plugs (expressed as fold change induction relative to PBS control) shows that Alboserpin significantly reduced endothelial permeability induced by FXa in vivo (Fig. 4E).

## DISCUSSION

Hematophagy is key to the reproductive success of blood-feeding arthropods, such as mosquitoes, and is an important link in pathogen transmission cycles. Salivary proteins from disease vectors have been shown to play a key role in modulating infection and viral replication, as well as host immune response (41–43). Blood-sucking arthropods counteract the host response to injury by expressing several bioactive compounds in their salivary glands that target wound healing, including platelet aggregation, blood coagulation, vasodilation, neutrophil function, angiogenesis, inflammation, and host immunity (44–53). Because inflammation and coagulation pathways are invariably linked (54, 55), FXa plays an important role in triggering proinflammatory responses by signaling through PARs, a type of G protein–coupled receptor found in various cell types, including endothelial cells, fibroblasts, keratinocytes, and smooth muscle cells, as well as platelets (56–60). Therefore, direct FXa inhibitors have the potential to prevent coagulation and inflammation. Two serine protease inhibitors that target FXa have been identified in the saliva of Aedes mosquitoes, Aedes aegypti FXa inhibitor and *A. albopictus* Alboserpin (19, 61, 62). Alboserpin is a potent salivary anticoagulant that displays competitive, reversible, and high-affinity binding to FXa. However, Alboserpin failed to inhibit several enzymes involved in hemostasis and inflammation, including serine protease, Kallikrein, granzyme B, matriptase, elastase, **α**-chymotrypsin, chymase, FXIa, FXIIa, plasmin, thrombin, trypsin, and cathepsin G. Alboserpin also binds heparin and membrane phospholipids, blocking prothrombinase activity in vitro. Consequentially, Alboserpin increases both prothrombin time and partial thromboplastin time in vitro and ex vivo (19). However, the role of Alboserpin in inflammation remains to be investigated.

In this work, we explored the anti-inflammatory properties of Alboserpin. We show that this salivary protein exerts strong anti-inflammatory activity in vivo and in vitro. FXa has been shown to induce proinflammatory cytokines similar to previous studies in atrial tissue (63), smooth muscle cells (26), RAW 264.7 macrophages (28), and human endothelial cells (35). In addition, in mouse fibroblasts, FXa has been shown to trigger the secretion of proinflammatory cytokines (22), as well as other inflammatory molecules like MCP-1 (MCP-1/CCL2) in HUVECs (23). Similar results have been reported in several cell types (64). Alboserpin inhibits FXa and its subsequent inflammatory and endothelial activation/disruption properties in vitro and in vivo. Our results suggest that secretion of Alboserpin may facilitate blood feeding by disrupting coagulation and by interfering with host inflammatory responses induced by FXa. Several examples from blood-feeding arthropods implicate salivary proteins in potentiating blood feeding by modulating immune responses in the host skin (24).

Alboserpin is a specific protein from female mosquito salivary glands. Because only female mosquitoes feed on blood and can transmit pathogens to their vertebrate hosts, this molecule could play a key role in arbovirus transmission. However, more research is needed to demonstrate the possible role of Alboserpin and pathogen transmission.

**FIGURE 5.**
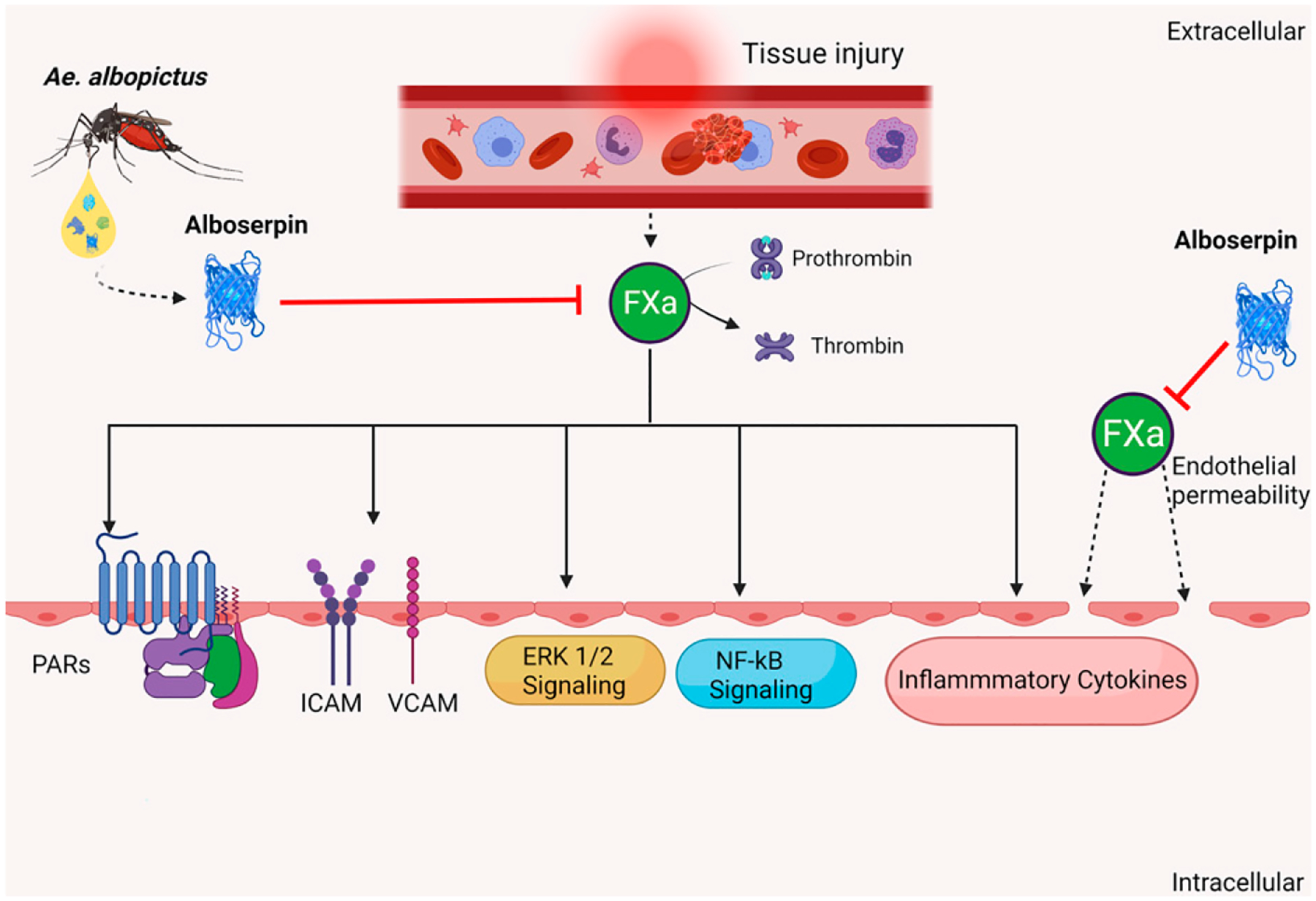
Schematic representation of the role of the Alboserpin effect in FXa-mediated cell signaling. During a mosquito bite, tissue injury causes the initiation of signaling through the extrinsic and intrinsic pathways that leads to the formation of FXa. FXa further converts prothrombin to thrombin, leading to clot formation. FXa triggers an inflammatory pathway in the endothelial cells by activating PAR receptors, ICAM and VCAM adhesion molecule expression, ERK1/2 signaling, NF-κB signaling, secretion of inflammatory cytokines, and disruption of endothelial cell barrier permeability. Mosquito A. albopictus salivary gland protein Alboserpin, a highly specific, strong inhibitor of FXa, prevents cleavage of PAR-2 by FXa, resulting in strong anti-inflammatory activity in vitro and in vivo.

Taken together, our results demonstrate that Alboserpin, the main salivary anticoagulant of the mosquito vector *A*. *albopictus*, also has strong anti-inflammatory activity in vivo and in vitro (Fig. 5). We also demonstrate that the anti-inflammatory mechanism of action of Alboserpin relies on the inhibition of FXa, leading to the inability of PAR signaling activation. The unique features of Alboserpin highlight the multifunctionality of salivary proteins and their possible implication in blood-feeding and pathogen transmission. This information highlights the complexity of the mosquito blood-feeding process and provides new evidence of multifunctionality of salivary molecules.

## Supplementary Material

Supplemental Material

## Abbreviations used in this article:

ATCC: American Type Culture Collection
FXa: factor Xa
HDMVECn: Dermal Microvascular Endothelial Cell; Normal, Human, Neonatal
NIAID: National Institute of Allergy and Infectious Diseases
NIH: National Institutes of Health
PAR: protease-activated receptor
qPCR: quantitative PCR
RT: room temperature
