# Supplementary material for: Alboserpin, the Main Salivary Anticoagulant from the Disease Vector Aedes albopictus, Displays Anti–FXa-PAR Signaling In Vitro and In Vivo
Source: Immunohorizons. Author manuscript; Available in PMC 2023 Dec 28. (PMC10753553; doi:10.4049/immunohorizons.2200045)
Supplement: Supplemental Material [file NIHMS1953982-supplement-Supplemental_Material.pdf]

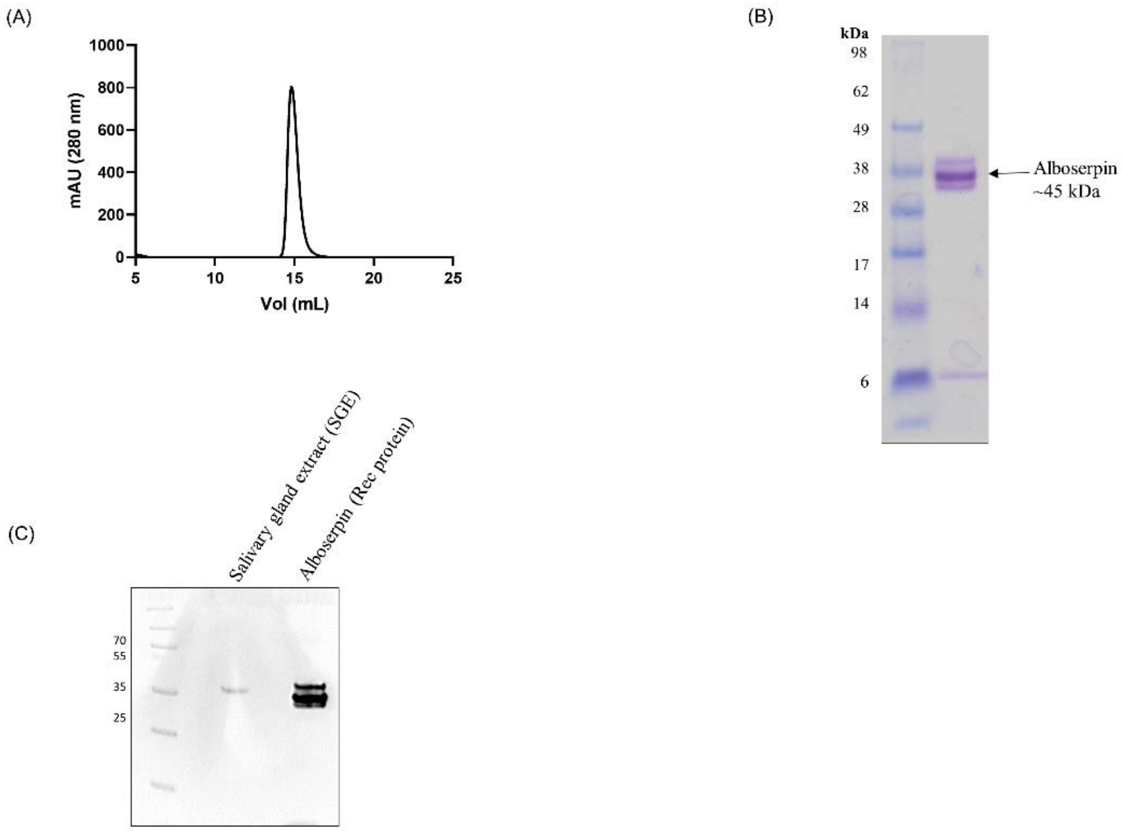

Supplementary Figure 1: Purification of recombinant Alboserpin and verification of Alboserpin IgG by western blot. A) Alboserpin was expressed in HEK293 cells, and the final step of purification was performed in a Superdex 200 increase 10/300 GL column. **B)** Coomassie-stained NuPAGE Novex 4–12% Bis–Tris gel electrophoresis of recombinant Alboserpin. **C)** Salivary gland extract (5 ug) along with recombinant alboserpin protein (500 ng) were resolved in NuPAGE Tris-bis protein gel. Proteins were transferred in PVDF membrane and further blotted against anti-alboserpin IgG (1:1000) as a primary antibody and HRP-anti-Rabbit as a secondary antibody. SuperSignal West Femto was used as a substrate to develop the bands.

Downloaded from http://journals.aai.org/immunohorizons/article-supplement/234075/pdf/ih_2200045_supplemental_1/ by guest on 21 December 2023

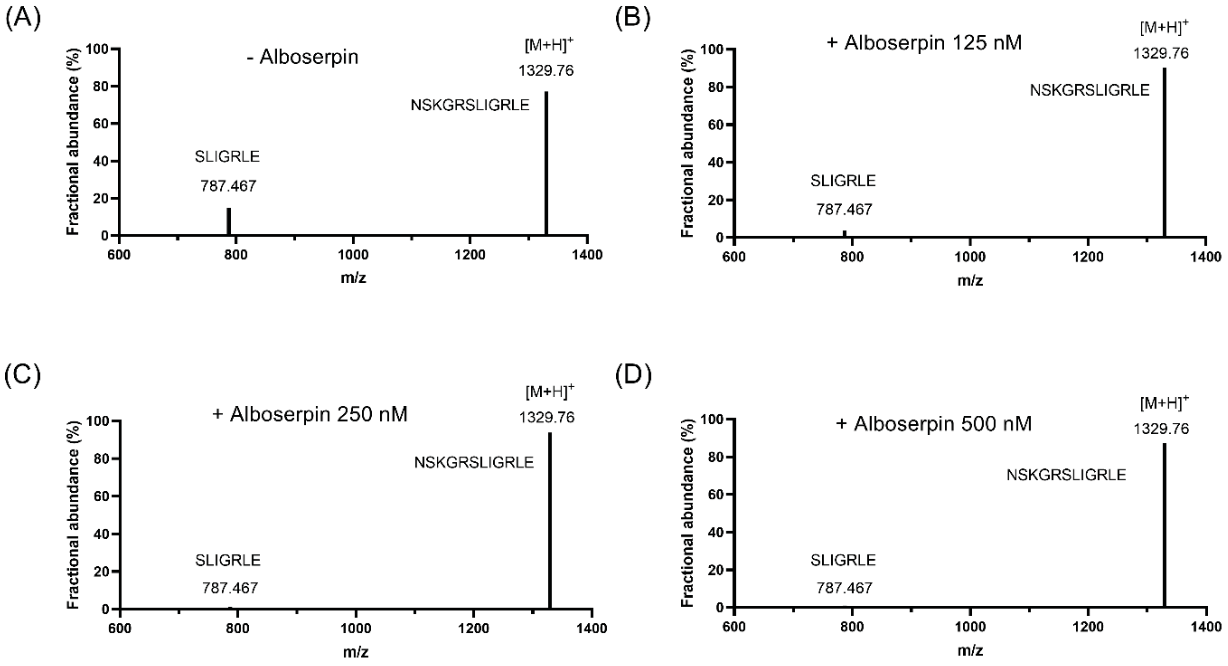

Supp. Fig. 2. ESI-MS analysis shows that PAR-2 peptide digestion by FXa is inhibited in the presence of Alboserpin. Deconvoluted mass spectra of full-length Par-2 peptide (NSKGRSLIGRLE, MH+ monoisotopic mass 1329.7597 Da) and FXa cleavage product (SLIGRLE, MH+ monoisotopic mass 787.4672 Da). FXa (25 nM) was incubated at 37°C for 15 minutes with different concentrations of purified Alboserpin (0, 125, 250, and 500 nM). PAR-2 (10 ug) was added in a final volume of 100 uL in PBS pH 7.4 and the reaction was incubated during 4 hours at 37°C. Par-2 incubated with FXa (A) or FXa+Alboserpin (B-D) were analyzed with a Q Exactive Plus Mass Spectrometer at 280k resolution. Mass spectra with less than 1% relative abundance, except for FXa cleavage product, were not shown in the figure.

Downloaded from http://journals.aai.org/immunohorizons/article-supplement/234075/pdfih_2200045_supplemental_1/ by guest on 21 December 2023

Supplemental Table 1: List of Primers used in this work.

Primer Name	Primer sequences (5'-3')
PAR 1- Forward	CCGCCTGCTTCAGTCTGTG
PAR 1- Reverse	GGCATTTGTTGCTTTTGATTCTCTGG
PAR 2- Forward	GTGGCACCATCCAAGGAACC
PAR 2- Reverse	TGTTTCAACTGTAACTCCTTTTCCA
PAR 3- Forward	GAAGCAGGAATATTATCTTGTTCAGC
PAR 3- Reverse	GGAGATGAAGTAATAGAGTTGGAAGG
PAR 4- Forward	CAGCCTGAGTGCAGTCATG
PAR 4- Reverse	TGAGGGCGTGCTGTCATC
NF-kB- Forward	CAGCAGGCAAACTCTCAGTCA
NF-kB- Reverse	CCAGATTGTCGCCGTTAATTTTT
VCAM- Forward	GGGAAGATGGTCGTGATCCTT
VCAM- Reverse	TCTGGGGTGGTCTCGATTTTA
ICAM- Forward	CGGATGAGAAGGTATTCGAGGT
ICAM- Reverse	CACCCACTTCAGGCTGGTTAC
GAPDH- Forward	CTCCTCTGACTTCAACAGCGA
GAPDH- Reverse	CCAAATTCGTTGTCATACCAGGA

Downloaded from http://journals.aai.org/immunohorizons/article-supplement/234075/pdf/ih_2200045_supplemental_1/ by guest on 21 December 2023
